# Three-dimension model of root canal morphology of primary maxillary incisors by micro-computed tomography study

**DOI:** 10.1016/j.heliyon.2024.e25890

**Published:** 2024-02-06

**Authors:** Lihua Lyu, Zhaohong Lin, Zheshan Zhao, Kezhen Wei, Hua Huang

**Affiliations:** a Department of stomatology, Children's hospital, Zhejiang University School of Medicine, National Clinical Research Center for Child Health, Hangzhou, Zhejiang, China; b Faculty of Odontology, University of Lorraine, Nancy, France; c Civil Aviation General Hospital, Beijing, China; d Department of Smile Angel Dental Hospital, Nanning, Guangxi, China; e Department of Pediatric Dentistry, College of Stomatology, Guangxi Medical University, Nanning, Guangxi, China

**Keywords:** Primary maxillary incisors, Root canal system, Morphology, Micro-computed tomography

## Abstract

The success of root canal treatment for deciduous teeth depends upon the shape of the root canal, among other factors. Despite this, there are limited reports on the use of high-resolution micro-CT to describe the root canal morphology of primary maxillary incisors. In this study, we aimed to create a three-dimensional (3D) digital model of the root canal morphology of primary maxillary incisors using microcomputed tomography (micro-CT). To provide a reference for the development of restorative posts for the primary maxillary incisors. Primary maxillary central and lateral incisors (n = 10 each) were analysed. Micro-computed tomography was used to conduct 3D analyses of the root canal system of the primary maxillary incisors. The canal volume and surface area of the primary maxillary central incisors were larger than those of the primary maxillary lateral incisors. The structural model index value was significantly lower in central incisors. At the cervical level and the interface between the cervical and middle one-third cross-sectional levels, the root canals of the primary maxillary lateral incisors were significantly rounder. The labio-palatal dimension and the diameters of the central incisors at the four different levels were significantly smaller than the diameter of the mesio-distal dimension. The taper of the central and lateral incisors gradually increased from the apical one-third to the cervical one-third in the labio-palatal dimension. The data obtained from the 3D analysis of maxillary incisors in this study will contribute to the design of root canal posts.

## 1 Introduction

Early childhood caries (ECC) is a common oral condition in children that often affects the primary incisors. When children seek dental care, the affected tooth is typically severely damaged with only the stump intact. In such cases, extraction of the affected tooth is a common clinical procedure [1,2]. However, premature loss of primary maxillary incisors can have adverse effects on children, including impaired pronunciation, reduced chewing function, development of abnormal tongue habits, subsequent malocclusion, and even impacts on mental health [3,4]. Therefore, there is an increasing focus on preserving and treating primary maxillary incisors. When the pulp of deciduous teeth is infected, the affected teeth are often repaired using endodontic treatment to allow natural exfoliation. There are various endodontic treatment methods for deciduous teeth, including indirect pulp capping, direct pulp capping, and pulpotomy, for preservation of the complete pulp. However, root canal treatment is preferred for patients with diffuse pulpal infection, pulpal necrosis, and periapical lesions of the deciduous teeth. Root canal treatment for deciduous teeth can eliminate pain, control inflammation, and promote healing by removing infected pulp inside the root canal, shaping the root canal, disinfecting the root canal, and filling the root canal with absorbable materials, to let deciduous teeth exfoliate naturally and allow the permanent teeth grow normally. The success of root canal treatment for deciduous teeth depends on the techniques, instruments, and materials used. The thicker root canals of deciduous teeth, different shapes, large apical foramina, and possible physiological and pathological root resorption require physicians to be careful when performing root canal treatment. Canals missed during treatment may lead to treatment failure when the inflammation cannot be controlled. Therefore, identifying the shape of the root canal in deciduous teeth is the key to successful root canal treatment.

In addition, repairing the residual roots and crowns of primary teeth with severe ECC poses significant challenges because of their propensity to fall. Although post crowns can be used to repair residual roots and crowns of permanent teeth with better clinical outcomes, there is currently no suitable retainer post available for primary teeth because of their physiological absorption. Several methods have been proposed to fix the root canals of primary teeth, including the use of fibre posts [1], resin short posts [5], and orthodontic steel wire bending [6]. However, these materials are unable to keep pace with the physiological absorption of primary teeth. Mizutani et al. [7] proposed a method for creating root canal posts using polylactic acid materials with bioabsorptive properties. Zou et al. [8] demonstrated the use of tooth-based materials to create root canal posts for primary teeth. In our earlier research [9], we fully dissolved a polylactic acid-absorbable root pipe pile in trichloromethane to create a polylactic acid film, which was then wrapped around the pile. This method closed the gap between the pile and root pipe wall, providing a synchronous absorption retention system for the polylactic acid absorbable root pipe pile. The sealing property of the post film system is similar to that of a glass ionomer cement, and it can withstand the required breaking force after repairing the residual root and crown [10,11]. Recently, a polylactic acid-absorbable post-membrane system has emerged as a potential method for restoring the residual roots and crowns of primary teeth. However, the polylactic acid absorbable root canal post mentioned above is made from absorbable nails used in orthopaedics, and its size and shape do not match the root canal of the primary incisors, leading to challenges in clinical use. Qualitative and quantitative data on the root canal system of primary maxillary incisors can provide a basis for the development of root canal pins that are absorbable and better fit the different shapes of deciduous teeth.

While studies on the root canal morphology of permanent teeth have advanced, primary teeth differ from their permanent counterparts in terms of external and internal morphologies [12]. Furthermore, the availability of complete primary tooth specimens is limited, which makes their collection challenging. Consequently, there are fewer data on the root canal morphology of primary teeth compared to permanent teeth, and the development of root canal morphology research has progressed from two-dimensional (2D) to three-dimensional (3D) methods and from qualitative to quantitative approaches. Previous studies have utilised techniques such as the clear tooth method [13] and the slice grinding method [14] to study the root canal morphology of primary teeth. However, these methods damage tooth specimens and cannot provide original information about the root canal, which can easily lead to measurement errors. Apical film methods [15], computerised tomography (CT) methods [16], and cone-beam CT (CBCT) methods [[17], [18], [19]] have also been reported for studying the root canal morphology of primary teeth. Although these methods have been successfully applied in the anatomical studies of tooth root canal systems, they have limitations. Root-tip films can only provide 2D images of 3D objects; thus, they fail to accurately represent the true shape of the research subject. CT scans are large and expensive and require high radiation doses. Compared with traditional medical CT, CBCT offers advantages such as shorter scanning times, lower radiation doses, and fewer image artefacts [20]. However, some studies have indicated that CBCT cannot provide clear root canal profiles and may distort the root canal tomography. With the advancement of computer 3D imaging technology in the medical field, micro-CT has been used for dental pulp experiments [21]. Owing to its high-resolution and non-destructive nature, micro-CT has become increasingly important in the study of root canal morphology, serving as a valuable tool for the qualitative and quantitative analysis of dental pulp [22,23]. Micro-CT addresses some of the limitations of previous methods. While Cumes et al. [24] used micro-CT to study the root canal morphology of primary molars, there are limited reports on the use of high-resolution micro-CT to describe the root canal morphology of primary maxillary incisors.

The objective of this study was to conduct a precise investigation of the root canal anatomy of primary maxillary incisors using micro-CT scanning and 3D reconstruction, to provide conditions for successful root canal treatment of primary maxillary inventors, and to provide a basis for the development of nickel titanium instruments and absorbable root canal posts for primary maxillary inventors.

## 2 Materials and methods

### 2.1 Research subjects

The Department of Anatomy at Guangxi Medical University obtained 20 primary maxillary incisors including 10 primary central maxillary incisors and 10 primary lateral maxillary incisors without any signs of absorption, from children aged 3–5 years of age from the Anatomy Teaching and Research Office. The exclusion criteria were as follows: teeth with (a) fractured roots, (b) structural defects, and (c) open apical foramina. This study was approved by the Ethics Committee of Guangxi Medical University (approval number: Shen 20,210,148).

### 2.2 Processing and preservation

The soft and hard tissues adhering to the tooth surface were thoroughly cleaned and immersed in small glass bottles containing 10% formalin for preservation.

### 2.3 Micro-CT scanning and 3D reconstruction

The teeth were gently dried and positioned in a sample holder with the root perpendicular to the bottom. A Swiss-made micro-CT scanner (μCT100; SCANCO Medical, Bassersdorf, Switzerland) was utilised to scan the tooth specimens from the crown to the apex of the root, followed by 3D reconstruction. The scanning parameters were set at a pixel resolution of 30 μm and a layer thickness of 30 μm.

### 2.4 Analysis of anatomical parameters of primary maxillary incisors

Cross-sectional views were generated by moving the crown towards the root. The first enamel image represents the cross-sectional view of the cervical root (C, Cervical), and the first image displaying a complete apical foramen represents the apical foramen (A, Apical). The root canal, from the cervical root to the apical foramen, was divided into three equal segments: the cervical one-third, middle one-third, and apical foramen. Four cross-sections were examined: the root cervical section (C), interface between the cervical one-third and middle one-third (CM), interface between the middle one-third and apical foramen one-third (MA), and apical foramen section (A).

#### 2.4.1 Root canal length, volume, surface area, and structure model index (SMI)

As micro-CT scanning was continuous without intervals, the number of layers between the section where the enamel appeared and the section where the first complete apical foramen appeared was recorded. The root canal length in this experiment was measured by multiplying the number of layers between the two points by 30 μm [25].

The SMI, an additional parameter used to describe the geometry of 3D objects, was initially proposed by Hildebrand et al. to characterise the structural features of the bone trabeculae. It is calculated using formula 6 * ((S' *V)/S^2^), where S and S′ represent the surface area before and after expansion, respectively, and V represents the initial volume [26]. Ideally, the SMI values should be 0, 3, and 4 for the disc, rod, and ball trabecular bone morphologies, respectively. In recent years, SMI has been used to assess the geometric shape of root canals, with higher values indicating a more rounded overall shape [27].

#### 2.4.2 Circumference, area, and roundness of each root canal cross-section

The circumferences and areas of the four root canal cross-sections were measured using micro-CT. Roundness, which represents the shape of a cross-section, was calculated using the formula: 4 *π * A/P^2^, where A is the area and P is the circumference [28]. The roundness values range from 0 to 1, with values closer to 1 indicating a shape closer to a circle.

#### 2.4.3 Labial-palatine diameter and mesio-distal diameter of each root canal cross-section

The micro-CT images of each tooth were individually examined to identify the corresponding cross-section, and the labial-palatine and mesio-distal diameters of each cross-section were measured using Mimics 17.0 image analysis software (Materialise, Leuven, Belgium).

#### 2.4.4 Taper of the root canal in three segments

The labial-palatine and near-distal tapers of the root canals in the cervical, middle, and apical foramen one-third were calculated based on the root canal diameters obtained from the four measured sections. The calculation formula is as follows: (D - d)/H, where D represents the diameter of the root canal in the crown section, d represents the diameter of the root canal in the root section, and H represents the vertical height between the two cross-sections [29].

### 2.5 Statistical analysis

Statistical analysis was conducted using SPSS 20.0 software. The root canal length, surface area, volume, SMI, and roundness of different cross sections of the primary central and lateral maxillary incisors were compared using a two-sample *t*-test. The buccal and proximal distal diameters of different root canal cross sections within the same group were compared using a paired *t*-test, whereas the tapering of different root canal segments in the same direction was compared using analysis of variance (ANOVA). The significance level was set at *p* < 0.05.

## 3 Results

### 3.1 Morphological observation results

#### 3.1.1 Morphology of primary central maxillary incisor

**Fig. 1:**
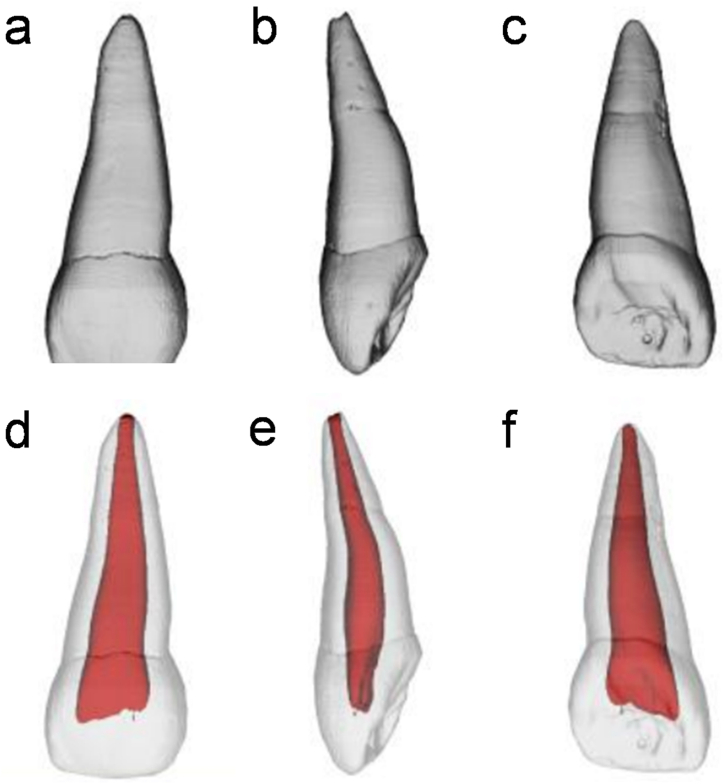
Exemplary three-dimensional models of primary maxillary central incisors (a–c) and their corresponding root canal morphology (d–f). Generally, the roots of the primary central incisors exhibit a wide and flat shape, featuring a single root canal without any apical ramifications or lateral canals.

The root of the primary central incisor is curved towards the lip side by approximately one-half or one-third and appears flat, wide, and slightly distal. Some teeth also exhibited a shallow longitudinal groove on the labial-palatal side (Fig. 1a–f).

#### 3.1.2 Morphology of the primary lateral maxillary incisor

**Fig. 2:**
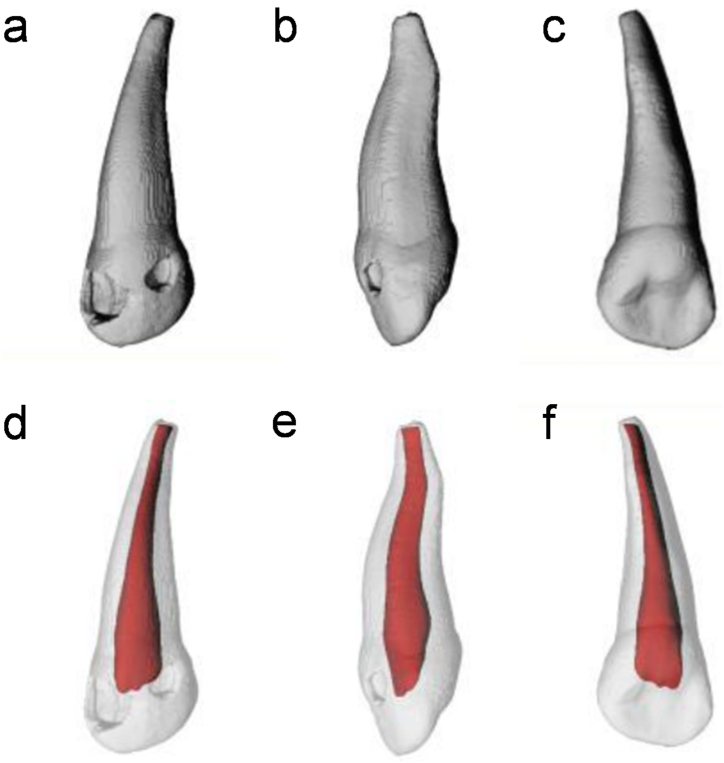
Exemplary three-dimensional models of primary maxillary lateral incisors (a–c) and their respective root canal morphology (d–f). Generally, the roots of the lateral incisors are narrow and thick, hosting a single canal without any apical ramifications or lateral canals.

The root tip of the primary maxillary incisor was curved towards the lip by approximately one-third, appearing narrow, thick, and slightly oblique to the distal side (Fig. 2a–f).

#### 3.1.3 Comparison of morphology between primary central maxillary incisor and primary lateral maxillary incisor

**Fig. 3:**
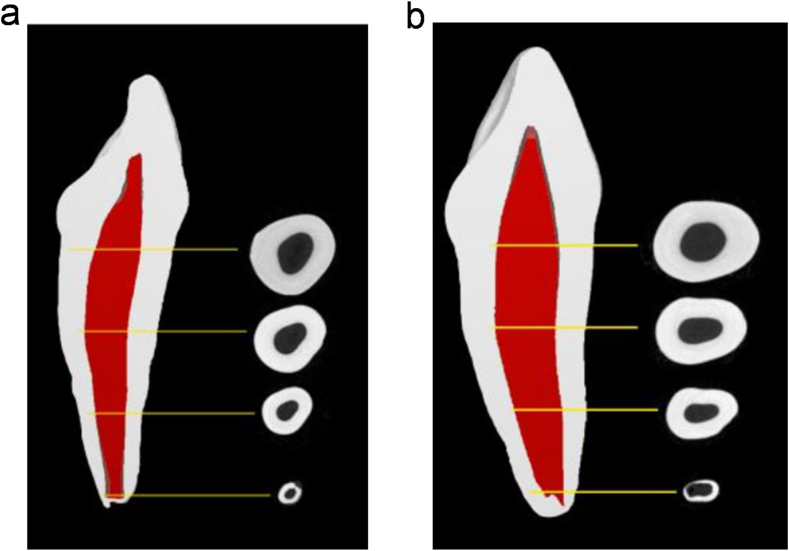
Three-dimensional reconstruction of the pulp cavity of primary central incisors (a) and lateral incisors (b), which showcases the variations in the two-dimensional configuration of the root canal at different levels of the root.

According to 3D models of the 20 primary maxillary incisors, the root surface adjacent to the primary maxillary incisor displayed an S-shaped curvature. All specimens had a single root canal, and no apical ramifications or lateral canals were observed. High consistency between the root canal morphology and dental roots was maintained. The cross-sections of the same canal exhibited variations, ranging from round to oval or triangular shapes (Fig. 3a–b).

### 3.2 Results of the quantitative analysis

#### 3.2.1 The length, volume, surface area, and SMI of the primary central maxillary incisor and primary lateral maxillary incisor

No significant differences in tooth length were observed between the two groups (*p* > 0.05). However, significant differences were found in tooth volume, surface area, and SMI between the two groups (*p* < 0.05). In summary, the primary central maxillary incisors exhibited larger volume and surface area compared to the primary lateral maxillary incisors, while the SMI of the primary central maxillary incisors was smaller than that of the primary lateral maxillary incisors. Table 1 shows the length, volume, surface area, and SMI of the primary central and lateral maxillary incisors.

**Table 1:** Root canal length, volume, surface area, and SMI (x ± s).

	Root canal length (mm)	Volume	Surface area	SMI
Primary central maxillary incisor	9.43 ± 1.35	15.35 ± 5.41	43.93 ± 9.90	2.69 ± 0.41
Primary lateral maxillary incisor	8.91 ± 0.97	9.23 ± 4.37	31.08 ± 9.44	3.23 ± 0.28
*t* value	0.992	2.786	2.970	−3.497
*p-*value	0.334	0.012	0.008	0.003

SMI, structural model index.

#### 3.2.2 Roundness of the cross-section of the primary central maxillary incisor and primary lateral maxillary incisor

For both the primary central and lateral maxillary incisors, the shape varied across different sections, with roundness gradually decreasing from the root cervical region to the apical region, followed by an increase at the apical foramen. In each cross-section, the roundness of the primary lateral maxillary incisors exceeded that of the primary central maxillary incisors. Statistically significant differences were observed in the C and CM cross sections (*p* < 0.05). Table 2 presents the roundness of each cross-section.

**Table 2:** Roundness of each section of the primary maxillary incisor (x ± s).

	Roundness			
	C	CM	MA	A
Primary central maxillary incisor	0.82 ± 0.01	0.76 ± 0.04	0.63 ± 0.12	0.66 ± 0.15
Primary lateral maxillary incisor	0.85 ± 0.01	0.79 ± 0.03	0.70 ± 0.08	0.72 ± 0.04
*t* value	−7.905	−2.181	−1.550	−1.083
*p-*value	0.000	0.043	0.138	0.303

C, the root cervical section; CM, the interface between cervical one-third and middle one-third; MA, the interface between middle one-third and apical foramen one-third; A, the apical pore section.

#### 3.2.3 The diameter of cross-sections of the primary central maxillary incisor and primary lateral maxillary incisor

Within each cross-section, the labial-palatine diameter of the primary central maxillary incisors was smaller than the mesio-distal diameter, and these differences were statistically significant (*p* < 0.05). Conversely, the labial-palatine diameters of the primary lateral maxillary incisors were significantly larger than the mesio-distal diameters (*p* < 0.05). Table 3 lists the diameters of each cross-section.

**Table 3:** Diameter of each section of the primary maxillary incisor (mm)(x ± s).

		C	CM	MA	A
Primary central maxillary incisor	Labial-palatine	1.69 ± 0.22	1.16 ± 0.16	0.63 ± 0.19	0.36 ± 0.17
	Mesio-distal	2.37 ± 0.34	2.09 ± 0.20	1.58 ± 0.22	0.73 ± 0.28
	*t* value	−10.295	−13.373	−9.445	−3.812
	*p-*value	0.000	0.000	0.000	0.004
Primary lateral maxillary incisor	Labial-palatine	1.88 ± 0.22	1.43 ± 0.38	1.02 ± 0.38	0.60 ± 0.37
	Mesio-distal	1.66 ± 0.26	0.92 ± 0.17	0.52 ± 0.16	0.36 ± 0.16
	*t* value	3.334	5.843	5.985	3.385
	*p-*value	0.009	0.000	0.000	0.008

C, the root cervical section; CM, the interface between cervical one-third and middle one-third; MA, the interface between middle one-third and apical foramen one-third; A, the apical pore section.

#### 3.2.4 Taper of the cross-section of the primary central maxillary incisor and primary lateral maxillary incisor

In the group of primary central maxillary incisors, the labial-palatine taper gradually increased from the apical one-third to the cervical one-third, whereas the opposite trend was observed in the mesio-distal dimension (*p* = 0.000, *p* = 0.001). In the group of primary lateral maxillary incisors, there was no significant difference in the labial-palatine root canal taper (*p* > 0.05); however, a statistically significant pairwise difference was found in the mesio-distal dimension (*p* < 0.05). Table 4 shows the tapering of each root canal segment.

**Table 4:** Taper of each root canal segment (x ± s).

Root canal segment	Primary central maxillary incisor		Primary lateral maxillary incisor	
	Labial-palatine^a^	Mesio-distal^b^	Labial-palatine	Mesio-distal^c^
Cervical one-third	0.16 ± 0.05	0.08 ± 0.07	0.16 ± 0.09	0.25 ± 0.06
Middle one-third	0.17 ± 0.03	0.16 ± 0.05	0.14 ± 0.04	0.14 ± 0.04
Apical foramen one-third	0.09 ± 0.02	0.28 ± 0.13	0.14 ± 0.05	0.06 ± 0.02
*F* value	11.676	13.321	0.191	48.624
*p-*value	0.000	0.001	0.827	0.000

a Buccolingual dimension of primary central maxillary incisors. The *p*-values for comparisons between the cervical one-third and middle one-third, cervical one-third and apical foramen one-third, and middle one-third and apical foramen one-third are *p* = 0.400, *p* = 0.001, and *p* = 0.000, respectively.

b Mesio-distal dimension of primary central maxillary incisors. The *p*-values for the comparisons between the cervical one-third and middle one-third, cervical one-third and apical foramen one-third, and middle one-third and apical foramen one-third are *p* = 0.043, *p* = 0.003, and *p* = 0.047, respectively.

c Mesio-distal dimension of primary lateral maxillary incisors. The *p*-values for comparisons between the cervical one-third and middle one-third, cervical one-third and apical foramen one-third, and middle one-third and apical foramen one-third are *p* = 0.000, *p* = 0.000, and *p* = 0.000, respectively.

## 4 Discussion

### 4.1 Guidance for the experiment in the study

Premature loss of primary teeth can influence the timing and sequence of eruptions of permanent teeth, which develop later. Therefore, to manage dental diseases in children, it is crucial to preserve teeth through restoration after endodontic treatment [30]. Familiarity with the root and root canal morphology of primary teeth is vital to enhance the success rate of endodontic treatment. The results of this study offer valuable insights for the research and development of restorative posts for primary maxillary incisors. The samples in this experiment revealed the presence of a single root canal in primary maxillary incisors. The tooth root had an S-shaped appearance when viewed from the side, with the root apex curved towards the lip by approximately half to one-third. This curvature may provide space for the eruption of the corresponding permanent teeth. These findings are consistent with previous studies [13].

The surface area and volume of the primary central maxillary incisors were significantly greater than those of the primary lateral maxillary incisors. This indicates that the primary central maxillary incisors possess a larger overall size compared to the primary lateral maxillary incisors. The SMI value, originally introduced by Hildebrand et al., describes the morphological structure of trabeculae as either dish-shaped (SMI = 0) or rod-shaped (SMI = 3) bone. An SMI value of 0 represents an ideal disk object, whereas a value of 3 indicates an ideal rod object [31]. In the present study, the mean SMI of the primary central maxillary incisor was 2.69, suggesting a conical root canal system. In contrast, the SMI value of the primary lateral maxillary incisor was 3.23, indicating a cylindrical root canal compared to that of the central incisor. These findings aligned with the shape of the root canal and the roundness observed in different sections. The roundness of the cross-section was used to represent the shape of the root canal. The results revealed that the same canal of a tooth exhibited various shapes at different horizontal cross-sections, which increased the challenge of root canal cleaning and filling [32]. However, both manual filing and rotating instruments have circular cross-sections that do not conform to the original shape of the root canal, leaving some areas inadequately prepared. The labial-palatine diameter of the primary central maxillary incisor was smaller than the mesio-distal diameter, whereas the opposite was true for the primary lateral maxillary incisor. This difference was also evident in 3D models. These findings differ from the research results of Long et al. [33], who used the radiovisiography (RVG) digital imaging method to study the root canal morphology of primary teeth (including eight central and lateral incisors), and found that the mesio-distal diameters of the primary central and lateral maxillary incisors were larger than the labial-palatine diameters. These disparities may arise from differences in sample sources and research methods. Micro-CT provides more accurate and reliable data than does RVG.

Based on the labial-palatine diameter, mesio-distal diameter, and taper of the three segments across the four cross-sections of the root canal in this study, it is evident that the standard 0.02 taper instrument has a taper that is too small for effective root canal preparation in primary maxillary incisors. Therefore, variable-taper instruments are recommended based on the taper measurement results of different parts of the root canal. The results of this study demonstrate that if the root canal instrument was selected based on the labial-palatine orientation, it would result in uncleaned areas on the mesio-distal surface. Conversely, if the mesio-distal orientation is used as a reference, excessive dentin removal occurs on the labial-palatine surface, and the same holds true for the primary lateral maxillary incisor.

Large, tapered nickel-titanium instruments have significantly improved mechanical root canal preparation [34]. However, these instruments are inadequate for cleaning oval or flat root canals and are prone to excessive dentin removal, leading to root fractures.

For permanent teeth, the length of the root canal post is typically half or two-thirds of the root length. However, in the past, to avoid impacting root resorption of the primary maxillary incisors, fibre, resin stub, and metal posts were only placed at one-third of the cervical portion of the root, which provided insufficient retention and stability. Although bioabsorbable materials are currently available, no root canal posts have been specifically designed for primary teeth. Instead, absorbable bone nails are used to shape a specific form [9], which does not guarantee proper adhesion of the post to the root canal wall. Therefore, the data obtained in this study will aid in the utilisation of bioabsorbable materials for the production of root canal posts for primary maxillary incisors. Because the root canal diameters of primary teeth vary in different horizontal planes, root canal posts should have a certain taper. Additionally, the labial-palatal diameters of the primary central maxillary incisor were larger than the mesio-distal diameters, while the opposite was true for the primary lateral maxillary incisor, suggesting that the root canal posts for the primary central maxillary incisor should differ from those of the primary lateral maxillary incisor.

### 4.2 Clinical application of the study results

Given that we identified that shape of the root canal of the primary lateral maxillary incisors differs from that of the primary central maxillary incisor and that the buccolingual diameter of the root canal in the primary central maxillary incisor is smaller than the mesio-distal diameter, and the opposite is true of the primary lateral maxillary incisors, our results highlight the importance of considering the taper measurements obtained from different sections of the root canal when selecting taper instruments. This will improve the efficiency and success rate of root canal treatments in deciduous anterior teeth. In addition, micro-CT scanning offers valuable insights into the development of absorbable root canal posts for deciduous teeth. Therefore, the results obtained in this study will be instrumental in advancing the design of restorative posts for primary maxillary incisors.

### 4.3 Limitations of the study

In this experiment, we only studied the canal morphology of 10 primary central maxillary incisors and 10 primary lateral maxillary incisors. Therefore, the small sample size was a limitation of this study. The small sample size was mainly due to the fact that complete primary maxillary incisors are difficult to find, let alone those with complete and unabsorbed roots. In this study, we provide root morphology and data on the primary central maxillary incisors and primary lateral maxillary incisors, which are consistent with those reported in relevant studies. This further validates the root canal morphology of primary maxillary incisors. Moreover, the high cost and lengthy scanning time of the micro-CT equipment restrict its clinical application, thereby limiting its use to in vitro experiments. Future studies should consider increasing the sample size to supplement the data obtained from this study. In addition, 3D finite element analysis can be employed to develop finite element models for post-core restoration of the primary central and lateral maxillary incisors, enabling the analysis of the influence of different diameters and depths of root canal posts on the stress distribution in the root tissue, which will promote the development of root canal treatment for deciduous anterior teeth, the clinical application of engine-driven nickel-titanium instruments, and the development of absorbable root canal pins for deciduous anterior teeth to enable their application in the repair of residual roots and crowns of deciduous anterior teeth as early as possible.

## 5 Conclusions

In conclusion, taper instruments should be selected according to taper measurements in different parts of the root canal to promote the development of engine-driven nickel-titanium instruments. The reconstruction of the root canal system of primary maxillary incisors will help paediatric dentists and other practitioners develop a better understanding of their morphology, to improve the efficiency and success rate of root canal treatment. In addition, our findings provide a basis for developing absorbable pins for deciduous teeth for better outcomes.

## Ethics approval

This study was approved by the Ethics Committee of Guangxi Medical University (approval number: Shen 20,210,148).

## Funding

This work was supported by the Clinical Research Fund for Oral Health Promotion and Oral Medicine Development of the Western Region of the Chinese Association of Stomatology (No. CSA-W2012-01).

## Data availability statement

Data included in article/supp. Material/referenced in article.

## CRediT authorship contribution statement

**Lihua Lyu:** Writing – review and editing. **Zhaohong Lin:** Formal analysis, Resources. **Zheshan Zhao:** Project administration. **Kezhen Wei:** Project administration. **Hua Huang:** Methodology, Conceptualization.

## Declaration of competing interest

The authors declare that they have no known competing financial interests or personal relationships that could have appeared to influence the work reported in this paper.
